# A concise review of glycerol derivatives for use as fuel additives

**DOI:** 10.1016/j.heliyon.2023.e13041

**Published:** 2023-01-18

**Authors:** A.L. Olson, M. Tunér, S. Verhelst

**Affiliations:** Division of Combustion Engines, Department of Energy Sciences, Lund University, SE-244 30, Lund, Sweden

**Keywords:** Biodiesel, Glycerol, Glycerol *tert*-butyl ether, Glycerol valorization, Oxygenated fuel additive, Solketal, Triacetin

## Abstract

Due to renewable fuel mandates worldwide, the increase in biodiesel production has caused an oversupply of low-cost glycerol on the markets, which can negatively affect the sustainability of the biodiesel industry as a whole. In order to avoid that scenario, the transformation of glycerol into value-added products has been investigated, and the production of additives for internal combustion engine fuels is one good example of glycerol valorization. The present work presents a summary of the literature describing the most important chemical pathways through which glycerol can be converted into fuel additives, to be subsequently mixed with either gasoline, biodiesel, or diesel fuel. The focus is on the three major categories, namely glycerol acetals/ketals, ethers, and esters (acetates). Moreover, the effectiveness of the different glycerol-derived compounds is illustrated through several examples from the literature. Finally, a few research gaps on the topic are identified and suggestions for future work are described.

## Highlights

• Literature review of glycerol-derived compounds that can be used as fuel additives.
• Covers the main types of glycerol-derived compounds: acetals/ketals, ethers, esters.
• A review of the usage of such compounds in gasoline, biodiesel, and diesel fuels.
• Focus on the exhaust emissions from internal combustion engines.

## Abbreviations

AET: Atmospheric Equivalent Temperature
AF: Aluminum Fluoride Three Hydrate
BGEs: Butyl Glycerol Ethers
CFPP: Cold Filter Plugging Point
CFR: Cooperative Fuel Research
DAG: Diacetylglycerol (diacetin)
DMS: Dimethyl Sulfate
EPA: Environmental Protection Agency
FAGE: Fatty Acid Glycerol Formal Ester
FAME: Fatty-Acid Methyl Esters
GBA: Glycerol Butyl Acetal
GBK: Glycerol Butanone Ketal
GC: Glycerol Carbonate
GDMEs: Glycerol Dimethoxy Ethers
GTME: Glycerol Trimethoxy Ether
GEA: Glycerol Ethyl Acetal
GHK: Glycerol Isohexanone Ketal
GIPE: Glycerol Isopropyl Ether
GTBE: Glycerol *Tert*-Butyl Ether
HRR: Heat Release Rate
LCA: Life Cycle Analysis
MAG: Monoacetylglycerol (monoacetin)
MON: Motor Octane Number
MTBE: Methyl *Tert*-Butyl Ether
NEDC: New European Driving Cycle
NO_x_: Nitrogen Oxides
OECD: Organization for Economic Co-operation and Development
PGEs: Propyl Glycerol Ethers
PM: Particulate Matter
RED: Renewable Energy Directive
RFS: Renewable Fuel Standard
RME: Rapeseed Methyl Ester
RON: Research Octane Number
RSM: Response Surface Methodology
SIPE: Solketal Isopropyl Ether
SME: Solketal Methyl Ether
STBE: Solketal *Tert*-Butyl Ether
TAG: Triacetylglycerol (triacetin)
TAME: Tert-Amyl Methyl Ether
TBA: *Tert*-Butyl Alcohol (Tert-Butanol)
THC: Total Hydrocarbons
TPGE: Tri-Propyl Glycerol Ether

## 1.0 Introduction

In the last several years, environmental issues related to air pollution and greenhouse gas emissions, along with concerns about energy security and the depletion of fossil fuels, have prompted governments around the world to implement mandates requiring the use of renewable energy sources. More specifically to the transportation sector, the mandates require that conventional fuels be gradually replaced by biofuels, such as ethanol and biodiesel. As an example, in the European Union, the 2009 Renewable Energy Directive (“RED”) (E.U. Directive 2009/28/EC) [1], aimed to increase the share of renewable energy use from 8.5% in 2005 to 20% by 2020. Subsequently, in December 2018 a new legislative framework was adopted under EU Directive 2018/2001 (the so-called “RED II”) [2], which is to be fully implemented by 2030. This new mandate requires that the minimum consumption targets of renewable energy are increased to at least a 32% overall share and a 14% share in the transport sector [3]. Moreover, in the United States, the Environmental Protection Agency’s (EPA) Renewable Fuel Standard (RFS) was created in 2005, requiring that the transportation fuel contained minimum annual amounts of renewables, to be increased each year [4]. In 2007, the program was expanded to achieve a long-term goal of 36 billion gallons (136.26 billion liters) of renewable fuels by 2022. However, according to a report by the U.S. Government Accountability Office, the program is unlikely to meet its goal of reducing greenhouse gas emissions due to insufficient production of advanced biofuels [5].

Biodiesel has become the leading biofuel in the European Union. As an example, in Europe, between the years 2000 and 2012, the production of biodiesel increased from 15 to 430 thousand barrels per day [6]. A 2014 report by the European Commission indicated that the biodiesel production capacity had increased to about 26.3 billion liters, with an annual production of about 10.5 billion liters, representing 40% of the total capacity [7]. According to data published by the Organization for Economic Co-operation and Development (OECD), global biodiesel production is projected to increase from 36 billion liters in 2017 to 44 billion liters by 2028, when the EU is expected to remain the world’s major producer [3]. Finally, it is estimated that the global biodiesel market will grow to approximately 63 million tons by 2030 [8].

**Fig. 1:**
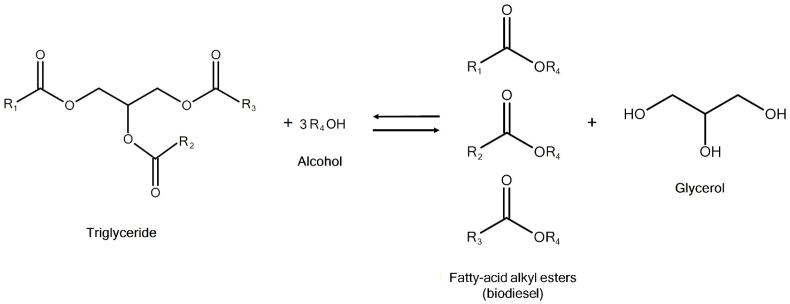
The transesterification reaction to produce biodiesel.

Chemically, biodiesel is defined as the mono-alkyl esters of long-chain fatty acids [9]. It is traditionally produced by the transesterification of triglycerides (that is, vegetable oils and/or animal fats) with an alcohol and a proper catalyst (Fig. 1). Methanol (methyl alcohol) is typically used, in which case the products are called fatty-acid methyl esters (FAME) [10]. The purpose of the transesterification reaction is to improve the properties of the original oil, such as lowering its viscosity, to make it suitable to be used as fuels for compression-ignition (diesel) engines. According to the stoichiometry of the transesterification reaction, 1 mol of triglyceride reacts with 3 mol of alcohol, producing 3 mol of fatty acid alkyl esters and 1 mol of glycerol, which corresponds to about 10 wt% of the biodiesel produced [[11], [12], [13]].

Consequently, the rapid growth of the biodiesel industry has caused an unavoidable oversupply of glycerol (a “glycerol glut”) on the markets. Because of that, the supply of glycerol is projected to grow faster than the demand for its traditional uses (e.g. in foods, cosmetics, and pharmaceuticals); the surplus is being consistently added to a relatively stable demand [14]. Globally, since 1995, there has been an oversupply of glycerol on the market, which keeps increasing due to the increased biodiesel production. Currently, it is estimated that the production of glycerol is six times higher than its demand [15]. A natural consequence of this glycerol oversupply is a sharp drop in the glycerol prices, and this has become a burden to the biodiesel industry.

Therefore, it has become necessary to find alternative ways of utilizing the glycerol from biodiesel production and turning it into valuable products in order to avoid market saturation and to ensure the sustainability of the biodiesel industry as a whole. Consequently, innovative processes have been investigated and developed to convert that glycerol into valuable chemicals, a topic commonly called glycerol valorization [[16], [17], [18], [19]]. As recent examples of glycerol valorization, it is worth mentioning the Epicerol® technology developed by Solvay [20], the production of bio-1,2-propanediol (bio-propylene glycol) by BASF and Oleon [21,22], and the industrial production of solketal-based Augeo™ SL 191 by Rhodia [23].

The production of fuel additives is thus one of the most important chemical routes used for glycerol valorization, since fuels are consumed in huge amounts worldwide. Such additives can be added to gasoline, biodiesel, and diesel fuel to improve their physicochemical properties and to minimize the emission of pollutants, by promoting cleaner combustion.

The present work aims to provide a concise yet broad review of the main chemical pathways that can be used to turn glycerol into various types of fuel additives, for either gasoline, biodiesel, or diesel fuel applications. The three major chemical product categories used as additives are glycerol ethers, acetals/ketals, and esters (acetates). Yet, there is a relative lack of studies in the literature covering these three categories at the same time as most of them only deal with a single type of compound. The focus is on linking the different chemical pathways together with practical applications, by analyzing the impact of the additives on the physical properties of the fuel blends and on the performance and emissions of engines fueled by such blends.

In the next paragraphs a brief description of glycerol and its properties is presented, followed by a general overview of the different types of fuel additives that can be synthesized from it.

## 2.0 Glycerol

Glycerol (1,2,3-propanetriol, commonly known as glycerin or glycerine) is a clear, colorless, odorless, sweet-tasting, hygroscopic, viscous liquid at room temperature. Its boiling point is 290 °C at atmospheric pressure and its freezing point is ca. 18 °C. It is an example of a polyalcohol, featuring a three-atom carbon chain with a hydroxyl group attached to each carbon. These groups make it completely miscible with water, methanol, ethanol, and the C3–C5 aliphatic alcohols. However, it is virtually insoluble in hydrocarbons [14]. Glycerol also has a very low toxicity and is biodegradable.

Even though a few authors have reported diesel engine operation running on glycerol [24,25], the direct utilization of it as a neat fuel is precluded by its physicochemical properties such as very high viscosity, high melting point, low heating value (16 MJ/kg), and high autoignition temperature (370 °C) [26]. Moreover, the polymerization issues of glycerol, along with its tendency to form propenal (also known as acrolein) during combustion further complicates the issue, as this is a toxic compound that causes irritation on the skin, eyes, and nasal mucosa. For that reason, for engine applications, glycerol has to be converted into compounds that can be mixed with fuels such as gasoline, biodiesel, or diesel.

As stated above, the production of fuel additives is one of the most important examples of glycerol valorization and it can be done by using different chemical routes. Regardless of the conversion method used, the many glycerol-derived additives can serve different functions, depending on the base fuel with which they are blended [27]. These different functions can be broadly categorized as gasoline octane boosters, biodiesel cold-flow improvers, and diesel fuel oxygenate (to decrease particular matter—PM—emissions).

A number of fuel additives—for gasoline, biodiesel, and diesel fuel—can be obtained from glycerol through different chemical pathways, the following of which have particularly been studied by several authors, namely, acetalization, etherification, and esterification. As an example of such versatility, a 2003 patent by Puche [28] describes the procedure of producing glycerol acetals, ketals, and acetates via the reaction of glycerol with aldehydes, ketones, acetic acid or methyl or ethyl acetate, with the goal of adding these compounds to biodiesel, in order to improve its properties at low temperatures. The patent explicitly describes the production of solketal, glycerol formal, and triacetin, obtained by reacting glycerol with acetone, formaldehyde, and methyl acetate, respectively.

In the following sections, these three chemical pathways are described and the literature reporting the use of the described additives in engines is reviewed.

## 3.0 Glycerol acetals/ketals

**Fig. 2:**
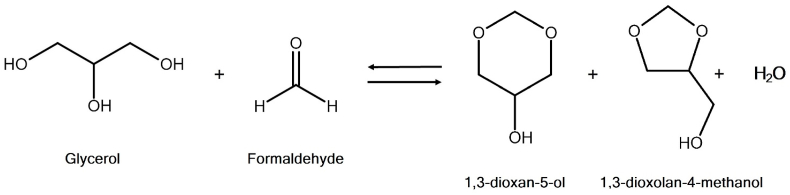
The acetalization of glycerol with formaldehyde.

Fuel additives can be obtained from the acetalization of glycerol with an aldehyde, over an acid catalyst, to produce acetals. An example of a glycerol acetal is the so-called glycerol formal, the product of the reaction between glycerol and formaldehyde (Fig. 2). It is a compound comprised of two cyclic isomers: 1,3-dioxan-5-ol and 1,3-dioxolane-4-methanol. This mixture of five- and six-membered cyclic isomers is a characteristic of the reaction of glycerol with aldehydes and ketones [29].

**Fig. 3:**
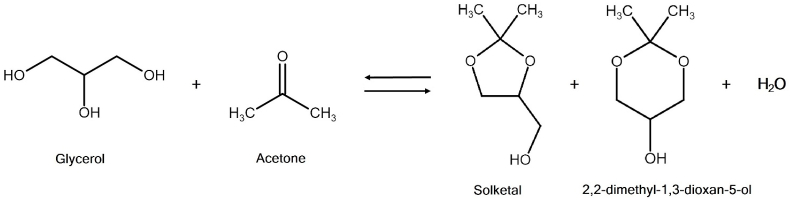
The ketalization of glycerol with acetone.

When glycerol is reacted with a ketone instead, the reaction is commonly called ketalization, and the product is called a ketal. A typical example is the reaction of glycerol with acetone (Fig. 3), yielding the five-membered ring compound 2,2-dimethyl-1,3-dioxolane-4-methanol, also known as solketal. In theory, besides solketal, this reaction also produces the six-membered ring 2,2-dimethyl-1,3-dioxan-5-ol. In practice, however, solketal is usually the only product, regardless of the catalyst used. This is due to the fact that the six-membered ring isomer is thermodynamically unstable due to the steric hindrance [30,31]. However, it is possible to produce the six-membered-ring acetal at high selectivities, as it has been reported in the literature. For example, a 2013 article by Khayoon and Hameed [32] reported the successful acetalization of glycerol with acetone under solventless conditions, yielding a final product comprising 74% of the five-membered ring solketal and 26% of the six-membered ring acetal.

Another study, by Khayoon et al. [33], investigated the acetalization of glycerol with acetone using multi-walled carbon nanotubes containing nickel nanoparticles as catalyst. Their results showed a glycerol conversion of 96% and selectivities of 72% and 28% towards solketal and the six-membered isomer 2,2-dimethyl-1,3-dioxan-5-ol, respectively.

The acetalization (or ketalization) of glycerol is usually a simple process that can be done under mild conditions at atmospheric pressure using standard acid catalysts, with high selectivity to products [34]. Also worth mentioning is the fact that the production of solketal directly from crude glycerol from biodiesel production—as opposed to purified glycerol—has been reported in the literature. For instance, Guidi et al. [35] investigated the continuous-flow acetalization of glycerol of different levels of purity using Amberlyst 36 and aluminum fluoride three hydrate (AlF3·3H2O, AF) as catalysts. Their results showed that AF is less sensitive to the presence of inorganic salts, a common contaminant of crude glycerol, and it was possible to achieve a conversion of 78% and 10 bar and 100 °C.

A 2018 article by Dmitriev et al. [36], investigated the possibility of using crude glycerol as a feedstock for preparing solketal. Additionally, their investigation also showed that glycerol of high purity can be produced from the hydrolysis of solketal. According to their method, the ketalization of crude glycerol with acetone was carried out at room temperature, using sulfuric acid as a catalyst, achieving an equilibrium conversion of 80% at 20–25 °C.

Another study, by Talebian-Kiakalaieh and Tarigi [37], demonstrated the use of synthesized hierarchical faujasite zeolite-supported heteropoly catalysts to obtain solketal using crude glycerol as feedstock. The authors showed that a maximum solketal yield of 97.87% could be obtained from crude glycerol at 100% conversion, at a temperature of 40 °C, in 2 h' reaction time. They concluded that the ketalization of crude glycerol to solketal can be carried out using that kind of catalyst and that a solketal yield above 86% and a glycerol conversion above 87% are possible, even in the presence of high concentrations of impurities, like water, methanol, and sodium chloride.

Another study investigating the continuous-flow ketalization of crude glycerol with acetone was carried out by Kuś et al. [38], using zeolites of different structures as catalysts. Their results showed a crude glycerol conversion of 85% and a selectivity to solketal of 98%, in a reaction time of 1 h, under mild reaction conditions. In addition, the authors described in detail the effect of glycerol impurities on the activities of the different kinds of catalyst, as a function of reaction time and catalyst structure.

The influence of solketal and glycerol formal on gasoline properties and performance was presented in a 2010 article by Mota et al. [39]. In their study, solketal and glycerol formal were blended with gasoline in concentrations of 1, 3, and 5 vol%. Both 100%-fossil gasoline and a gasoline containing 25 vol% ethanol were used, for a total of 12 different blends. The authors found that solketal was soluble in both gasolines, whereas glycerol formal was soluble only in the gasoline containing 25 vol% ethanol. Moreover, they determined that the addition of either solketal or glycerol formal to the gasolines did not change their distillation curves. Another finding was that the addition of solketal decreased the gum formation in both gasolines, whereas the addition of glycerol formal significantly increased gum formation. Finally, the authors measured the research octane number (RON) of the blends on a dedicated octane testing CFR engine and determined that solketal increased RON by up to 2.5 points, when mixed with the gasoline without ethanol. Glycerol formal, on the other hand, produced a slight decrease of 0.75 points in the octane number.

Glycerol acetals, including solketal, also feature anti-wear properties when mixed with fuels or lubricating oils. For example, in his 2012 patent, McDougall proposed a composition of engine lubricating oil containing 1,3-dioxolane-4-methanol [40]. A study by Samoilov et al. [41] focusing on the production of di-GTBE, solketal, and solketal *tert*-butyl ether (STBE, see Section 4.2), also investigated the anti-wear properties of these glycerol-derived compounds when mixed with heavy cycle oil, a low-viscosity hydrocarbon oil fraction. The tests were conducted according to ASTM D2266 [Standard Test Method for Wear Preventive Characteristics of Lubricating Grease (Four-Ball Method)]. Their results showed that solketal had the most significant influence on the anti-wear properties, when compared to STBE and GTBE. The authors ascribed this effect to the increased surface activity associated with the free hydroxyl group in the solketal molecule.

The effect of the addition of solketal to gasoline was investigated by Alptekin and Canakci, in their 2017 article [42]. For their experiments, a blend of 9 vol% solketal in gasoline was prepared and compared to 100% fossil gasoline. The fuel properties of the gasoline-solketal blend were analyzed and it was determined that the addition of solketal caused an increase in both the density and the flash point of the fuel blend. Additionally, the octane numbers were measured and the gasoline-solketal blend had a slightly higher research octane number (RON) than the standard gasoline (97 and 96.4, respectively). It is worth noting that the gasoline-solketal blend fulfilled the requirements of the EN 228 fuel standard [43]. Moreover, the presence of solketal was shown to decrease gum formation, in comparison with neat gasoline. Finally, vehicle emission tests were carried out on a chassis dynamometer at several steady-state speeds and engine loads. The results showed that the addition of solketal decreased the emissions of carbon monoxide (CO) and total hydrocarbons (THC), when compared to pure gasoline, but the emissions of nitrogen oxides (NO_x_) were 3.5% higher. The volumetric fuel consumption was slightly higher with the gasoline-solketal blend, which is expected, since solketal has a lower heating value than gasoline.

In spite of its qualities (such as renewability, good heating value, oxygen content, high cetane number), biodiesel suffers from poor flow properties at low temperatures, which can lead to problems such as fuel filter plugging. The use of glycerol-derived compounds is among the suggested solutions for the so-called winterization of biodiesel and glycerol-derived compounds, including acetals and ketals, have shown to be effective in improving the fuel’s cold-flow properties.

García et al. [44], in their 2008 study, evaluated the impact on physical properties of adding glycerol derivatives to biodiesel and biodiesel-diesel blends. Solketal was among the compounds tested and it was added in a 5 vol% concentration to both pure biodiesel (B100) and to a blend of 50 vol% biodiesel in fossil diesel fuel (B50). Their results showed that the addition of 5 vol% solketal in either B100 or B50 increased the density of the blends, causing it to be outside the maximum limit prescribed by the EN 590 standard (EU standard for diesel fuels) [45]. Additionally, the 5 vol% solketal addition to either fuel (B50 or B100) caused the flash point of the blend to be lower than the minimum values required (55 °C) by the EN 590 diesel standard. An improvement in both properties was observed when the hydroxyl group of the solketal molecule was replaced with an acyl group, after reacting it with acetic anhydride.

The effect of glycerol acetals on the pour point temperature of biodiesel was investigated in a 2010 study by Silva et al. [46]. They carried out the acetalization of glycerol with butanal, pentanal, hexanal, octanal, and decanal, to produce acetals of different chain lengths. The various acetals were then mixed with animal-fat-based biodiesel in 1 vol% and 5 vol% concentrations. Their results showed that the ability of an acetal to decrease the pour point temperature of biodiesel decreases with increasing chain length of the acetal’s molecule. Further, the acetal concentration of 1 vol% was insufficient to decrease the biodiesel pour point. In their study, the best result was obtained by mixing 5 vol% of the glycerol-butanal acetal in biodiesel, in which case a 5 °C reduction (13 °C vs 18 °C) in pour point temperature was observed.

De Torres et al. [47], in a 2012 study, produced glycerol ketals using a variety of linear, branched, and cyclic ketones, namely acetone, 2-butanone, cyclopentanone, 4-methyl-2-pentanone, and 3,3-dimethyl-2-butanone. Their goal was to select the most promising ketals and investigate their suitability as additives for biodiesel, focusing on cold flow properties, viscosity, and oxidation stability of the biodiesel-ketal blends. The glycerol ketals chosen for subsequent biodiesel blending were the ones derived from 2-butanone and cyclopentanone, due to their chemical structure and high production yields. Then, blends of these two ketals with biodiesel were prepared, in concentrations of 1, 5, 10, and 20 vol%. The largest decrease in cold filter plugging point (CFPP), 3 °C, was observed with the 20 vol% blend of the 2-butanone-derived ketal. While the cold flow properties of these biodiesel-ketal blends improved, their oxidation stability worsened and their ester content dropped below the 96.5% FAME content required by the European biodiesel standard, EN 14214. To improve the oxidation stability of the blends, the 2-butanone-derived ketal was esterified with either acetic or butyric anhydride, and the resulting compounds were blended with biodiesel. Finally, the 20 vol% blends achieved even larger decreases in CFPP, 6 °C, and much improved oxidation stability, when compared to blends with the non-esterified ketals.

In a patent from 2001, Delfort et al. [48] proposed a formulation for a diesel fuel capable of decreasing particulate matter (PM) emissions, containing acetals or ketals in concentrations ranging from 1 up to 40 vol%. The document also described the preparation of three different types of glycerol acetals, obtained by reacting glycerol with n-butyraldehyde (butanal), formaldehyde (methanal), and 1,1-diethoxyethane (acetaldehyde diethyl acetal). Finally, the patent described the PM emission results from a car on a New European Driving Cycle (NEDC) test, fueled by 5 vol% blends of the three acetals obtained according to the description above. Compared to the baseline fossil diesel figures, the decrease in PM emissions from the three acetals was between 19% and 21%.

A 2005 article by Jaecker-Voirol et al. [49] presented a comparison of several glycerol derivatives as blending components for diesel fuel. Among these components, two acetals are worth mentioning: glycerol butyl acetal (GBA) and glycerol ethyl acetal (GEA). The focus of their tests was to evaluate the impact on the exhaust emissions of blending the oxygenates into diesel fuel. The tests comprised two vehicles (Euro II- and Euro III-compliant) running on the NEDC driving cycle. The reduction in PM emissions from the Euro II car was around 21% for the 5 vol% GBA-diesel blend and 19% for the 5 vol% GEA-diesel blend. The impact of these same blends on the Euro III vehicle PM emissions was less pronounced (around 12% and 2% reductions, for the GBA and GEA blends, respectively).

In their 2014 investigation, Oprescu et al. [50] prepared (2-ethyl-2-methyl-1,3-dioxolan-4-yl)methanol, a glycerol ketal based on butane-2-one, and then esterified it with both methyl propionate and methyl hexanoate to produce the glycerol ketal esters (2-ethyl-2-methyl-1,3-dioxolan-4-yl)methyl propionate and (2-ethyl-2-methyl-1,3-dioxolan-4-yl)methyl hexanoate, respectively. Subsequently, samples of the mixtures of these three compounds with diesel fuel were prepared and analyzed. Their first aim was to study the effect of glycerol-derived compounds on the physical properties of the diesel blends, in concentrations of 1, 2, 5, and 7 wt%. Their results showed that the density and the viscosity of the diesel blends increased slightly with the increase in the ketal esters' chain length, i.e. the diesel blends with (2-ethyl-2-methyl-1,3-dioxolan-4-yl)methyl hexanoate were denser and more viscous than the diesel blends with (2-ethyl-2-methyl-1,3-dioxolan-4-yl)methyl propionate. Similarly, the flash point and the pour point of the diesel blends increased with increased chain length of the ketal ester. The cetane number of the blends was estimated based on the density and distillation curve of the blends, according to the standard ISO 4264 (‘Petroleum products — Calculation of cetane index of middle-distillate fuels by the four variable equation’). According to the results, the 1 wt% blends showed a slight increase in cetane number, when compared to neat diesel, and the increase was greatest with the ketal ester based on methyl hexanoate. Lastly, the authors evaluated the effect of the oxygenated blends on the performance and emissions from a 4-cylinder, 1.5-L light-duty diesel engine. Based on their results, compared to the neat diesel results, the blends of diesel with either (2-ethyl-2-methyl-1,3-dioxolan-4-yl)methanol or (2-ethyl-2-methyl-1,3-dioxolan-4-yl)methyl hexanoate slightly improved engine performance and decreased the emissions of unburned hydrocarbons, carbon monoxide, and smoke. On the other hand, the NO_x_ emissions were slightly increased.

## 4.0 Glycerol ethers

Ethers of glycerol have already been explored for several decades. In 1934, Evans and Edlund described a method for producing tertiary ethers of aliphatic polyhydric alcohols—such as glycerol—with tertiary-base olefins [51] and in 1941, Doelling found that certain glycerol mono- and di-ethers could be used as antiseptics [52]. Indeed, glycerol etherification has been extensively investigated to produce a wide variety of products, from food flavoring agents to solvents, surfactants, and fuel additives.

For fuel applications, glycerol is typically etherified by reacting it with an alkylation agent, such as alcohols or olefins (alkenes), in the presence of a strongly acidic catalyst, which can be either homogeneous (e.g. sulfuric acid) or heterogeneous (e.g. ion-exchange resins, zeolites, etc.). Several processes for the etherification of glycerol with ethanol, *tert*-butanol (*tert*-butyl alcohol, TBA), n-butanol, and higher alcohols have been proposed as well as the alkylation of glycerol with olefins (such as isobutylene).

The most commonly investigated glycerol ether, GTBE (glycerol *tert*-butyl ether), is introduced in the following paragraphs, together with its applications as a fuel additive. Then, other types of glycerol ethers are discussed.

### 4.1 GTBE (glycerol *tert*-butyl ether)

A well-known etherification reaction is the *tert*-butylation of glycerol, which replaces one or more hydroxyl groups by one or more *tert*-butyl groups, producing the so-called glycerol *tert*-butyl ethers (GTBE) [53,54]. Glycerol *tert*-butylation has been extensively investigated and industrial productions methods have been proposed [55,56]. This reaction is typically carried out when the alkylation agent is either isobutylene (isobutene) or *tert*-butanol. It is possible to achieve a combination of 100% glycerol conversion and very high selectivity (>92%) towards di- and tri-ethers (the GTBE components with best potential as fuel additives—see below) by using isobutylene as alkylation agent and ion-exchange resins as catalyst [57]. However, the process can be costly, isobutylene needs to be pressurized to be in liquid phase and it has low solubility in glycerol [58]. The use of TBA does not pose such problems and it also inhibits secondary reactions like isobutene oligomerization [59], but it creates water as a by-product, which can hamper the reaction by inhibiting catalyst activity [60].

**Fig. 4:**
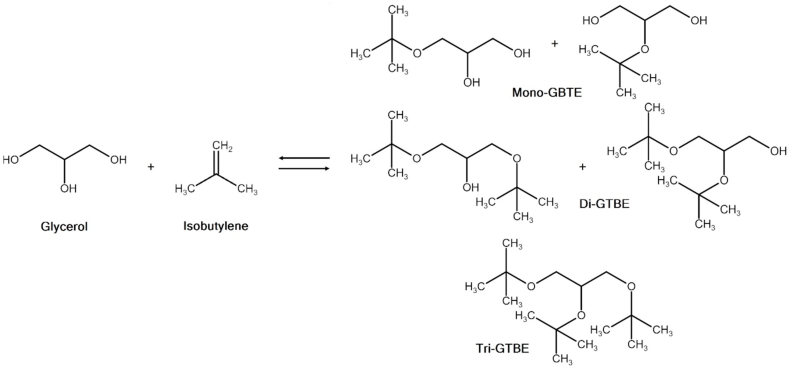
The etherification of glycerol with isobutylene.

GTBE is not a single compound, but rather a mixture of five component ethers, which are formed depending on the degree of etherification underwent by the glycerol molecule (i.e. depending on how many hydroxyl groups are substituted by a *tert*-butyl group). These five components are represented by three types of ethers: a monoether (mono-GTBE, representing two isomers), a diether (di-GTBE, two isomers), and a triether (tri-GTBE), all of which are shown in Fig. 4. Due to the poor miscibility of mono-GTBE in hydrocarbons, the *tert*-butylation reaction should be designed for high selectivity towards the higher ethers, that is, di- and tri-GTBE. The latter exhibits the best hydrocarbon solubility, but its synthesis can be difficult due to steric hindrance [41] and it is more expensive, since it consumes more alkylating agent.

As stated previously, glycerol ethers are versatile compounds with a potential to be used in many different applications. In particular, the tertiary butyl ethers of glycerol (GTBE) have been widely investigated as promising additives for gasoline, diesel, and biodiesel fuels. Furthermore, renewable GTBE (obtained from waste glycerol from biodiesel production) has the potential to increase the share of renewable energy used in the European transportation sector, helping achieve the EU Directive’s targets.

The potential of GTBE as a fuel additive partly stems from its versatility to serve different functions, depending on the base fuel to which it is blended, whether it be gasoline, diesel, or biodiesel. As an example, Bradin [61] proposed extensive applications for glycerol ethers, as additives for a wide variety of fuels, including gasoline, gasoline-alcohol blends, biodiesel, diesel fuel, marine diesel fuel, and jet fuel.

#### 4.1.1 GTBE as gasoline additive

Methyl *tert*-butyl ether (MTBE) is a gasoline octane booster and it is usually produced by etherifying methanol with *tert*-butanol or isobutylene. It has been used as a gasoline octane-boosting additive in many parts of the world, superseding tetraethyl lead. However, due to concerns about groundwater and soil contamination, many countries have been phasing out MTBE and replacing it with other gasoline oxygenates.

Chemically, GTBE can be considered the glycerol-equivalent of MTBE and, as such, it has been shown to be effective in increasing the octane rating of gasoline. Wessendorf [62] reported blending octane numbers between 112 and 128 (RON) for GTBE mixtures, making glycerol ethers suitable to be used as gasoline additives.

In their patents, Krasnykh et al. [63] and Versteeg and Wermink [64] described methods to produce GTBE to be used as octane booster.

The effect of GTBE on gasoline was reported in a recent study by Bozkurt et al., in a 2019 article [65]. In their study, six different GTBE mixtures, with varying amounts of mono-, di-, and tri-ethers were prepared by etherifying glycerol with isobutylene. Subsequently, each of these mixtures were blended with gasoline, at a concentration of 3.45 vol%. In addition, a blend of 3.45 vol% methyl *tert*-butyl ether (MTBE) in gasoline was also prepared. Their findings showed that the GTBE mixture containing the highest amount of the tri-ether (34 wt%) formed the most homogeneous solution with gasoline, when added at the 3.45 vol% concentration. Also, it increased the octane number (RON) of the gasoline by one point (from 95 to 96). This blend was then chosen for engine testing, and the results showed that GTBE is a good alternative to MTBE, as it resulted in comparable engine performance and fuel consumption, and similar exhaust emissions.

The second part of a comprehensive two-part study by Samoilov et al. [66] investigated the main physicochemical properties, as well as the gasoline-blending characteristics of di-GTBE (glycerol 1,3-di-*tert*-butyl ether) and also of a wide range of diol derivatives, comprising ethers, cyclic ketals, and O-ethers of solketal. These were: 2,2,4-trimethyl-dioxolane-1,3 (TMD); 2,2-dimethyl-dioxolane-1,3 (DMD); glycerol butanone ketal (GBK); glycerol isohexanone ketal (GHK); solketal isopropyl ether (SIPE); solketal *tert*-butyl ether (STBE); glycerol 1,3-di-isopropyl ether (di-GIPE); and glycerol 1,3-di-*tert*-butyl ether (di-GTBE). The authors' goal was to analyze the relationship between the compounds' molecular structure and their efficiency as gasoline oxygenates. For the pure oxygenates, their density, boiling and melting points, viscosity, and net heat of combustion were measured. Additionally, blends of such compounds with neat fossil gasoline were prepared (in concentrations of 1.0, 2.5, 5.0, 7.5, and 10.0 vol%). Subsequently, the blends' density, heating value, fractional composition, vapor pressure, research octane number (RON), motor octane number (MON), oxidation stability, gum content, and pour point were determined. According to the results, di-GTBE was the most effective octane booster among all derivatives, with average blending RON and MON values of 135 and 117, respectively. Furthermore, the authors found that, when compared to the usual gasoline oxygenates ethanol and MTBE, di-GTBE exhibited superior octane-boosting performance, based on the ratio of the increase of the blend’s antiknock index to the decrease in its heating value (due to oxygenate addition). However, as a drawback, their results also showed that the addition of all oxygenates had decreased the volatility of the base gasoline and distorted its distillation pattern. Further, this negative impact appeared to be worse in the case of the glycerol derivatives. Due to the fact that all-glycerol-derived additives decreased the vapor pressure and increased the final boiling point of the base gasoline, the authors recommended the addition of lighter fractions to it, in order to compensate for this negative effect.

#### 4.1.2 GTBE as biodiesel additive

As stated above, glycerol-derived compounds, such as GTBE, can be effective in improving biodiesel’s cold-flow properties. In his patents [67,68], Noureddini proposed a biodiesel formulation with improved cold-flow properties consisting of 12 wt% GTBE and 88 wt% methyl esters. With such a blend, he reported a viscosity reduction greater than 0.5 mm^2^/s at 70 °F (21 °C) and a decrease of 9 °F (5 °C) in cloud point, when compared to the biodiesel fuel without glycerol ethers. That patent also describes a process for the combined production of methyl esters and glycerol ethers, thus enabling the conversion of essentially all feedstock (triglycerides) into an improved biodiesel formulation.

Another patent, by Hillion et al. [69], proposed a similar approach, in which the totality of the by-product glycerol is etherified with isobutylene to form GTBE and the ethers are then mixed with the alkyl esters, resulting in an improved biodiesel consisting of 18 wt% glycerol ethers.

A study by Melero et al. [70], published in 2010, investigated the influence of glycerol derivatives on biodiesel quality parameters, against the requirements of the EN 14214 European biodiesel standard. The glycerol derivatives included: GTBE (comprised of 5 wt% mono-ethers, 55 wt% di-ethers, 38 wt% tri-ether, and 2 wt% of remaining di-isobutylene), a mixture of glycerol acetates (comprised of 6 wt% monoacetin, 45 wt% diacetin, 47 wt% triacetin, and 2 wt% of remaining acetic acid), neat triacetin, and neat solketal. These compounds were mixed with soybean biodiesel in different amounts and their influence on biodiesel properties was determined. The properties, as described by the EN 14214 standard [71], included: density, viscosity, pour point, cold filter plugging point (CFPP), and oxidation stability. The authors concluded that the best performance was achieved by the GTBE mixture, at a concentration of 10 g per 100 g of pure biodiesel. This blend fulfilled the majority of the EN 14214 parameters, except a few, such as the ester content (it was 87.3 wt%, below the 96.5 wt% required by the standard).

#### 4.1.3 GTBE as diesel additive

As described above, glycerol *tert*-butyl ethers, being comprised of components with branched molecules, have high octane ratings and are suited as gasoline additives. In spite of that, GTBE can also be used, in moderate amounts, as diesel oxygenate to reduce particulate matter emissions.

For example, in their 1994 patent, Kesling et al. [72] tested a number of blends of diesel-GTBE and diesel-biodiesel-GTBE on a heavy-duty engine, on an EPA transient test cycle. The results showed a PM reduction of up to 25.8% with a 5 vol% GTBE blend, compared to regular diesel fuel. Furthermore, a blend consisting of 5 vol% of an “improved” biodiesel (a mixture of 20 vol% GTBE and 80 vol% biodiesel) in diesel fuel decreased PM emissions by 13.2%, when compared to a blend of 5 vol% “regular” biodiesel in diesel fuel. Their results show the ability of GTBE to reduce diesel PM emissions, not only when added to diesel fuel, but also when added to the biodiesel which is going to be subsequently blended with diesel.

A 2003 study by Spooner-Wyman et al. [73] addressed the impact of both diesel-GTBE and diesel-biodiesel-GTBE blends on the exhaust emissions from a heavy-duty diesel engine. They reported reductions in PM emissions of 13.8% with a 15 vol% GTBE blend in diesel fuel, whereas the blend comprised of 4 vol% GTBE and 16 vol% biodiesel in diesel fuel gave a PM reduction of 16%, when compared to neat diesel fuel.

A 2008 article by Jaecker-Voirol et al. [74], presented the results of a study involving a number of glycerol derivatives to be used as diesel additives by proposing a new biodiesel formulation. The derivatives comprised glycerol ethers, acetals, and carbonates. Such compounds were blended with biodiesel (rapeseed methyl ester, RME) in different concentrations, then they were mixed with fossil diesel fuel and tested on two passenger cars (complying with the Euro II and Euro III legislation) over the New European Driving Cycle (NEDC). As a comparison, blends of RME in 5% and 10% in fossil diesel were also tested. According to their emission results, GTBE (made up of 13 wt% mono-ethers, 64 wt% di-ethers, and 22 wt% tri-ether) was the most interesting glycerol derivative; therefore, their final proposed biofuel formulation was comprised of 92.5% RME and 7.5% GTBE, also adding 1000 ppm ethyl-2-hexyl-nitrate to compensate for the lower cetane number. This biodiesel formulation was thus supposed to be blended with fossil diesel in a concentration of 5 vol%.

Exhaust PM reductions were also reported in recent studies by Di Blasio et al. [75] and Beatrice et al. [76], where they investigated the impact of diesel-GTBE blends on the emissions from a light-duty single-cylinder research diesel engine. Their results showed that, with a blend of 20 vol% GTBE in diesel fuel, PM emissions were reduced by up to 70% at medium to high loads, along with a slight increase in nitrogen oxides (NO_x_). At low loads, however, the GTBE blends increased the emissions of both carbon monoxide (CO) and total hydrocarbons (THC) by up to about 50%, most likely due to combustion inefficiency caused by GTBE’s low cetane number. At low loads, the addition of GTBE also caused an increase in aldehyde emissions.

Another work by Beatrice et al. [77], published in 2013, continued the testing of GTBE on the light-duty single cylinder diesel engine used in the studies described above. In their investigation, a mix of GTBE was prepared using isobutylene as alkylating agent and a novel catalyst based on perfluorosulphonic ionomer and having spherical silica as carrier. The GTBE mix thus obtained was comprised of 1.6 wt% mono-ethers, 39.2 wt% di-ethers, and 58.3 wt% tri-ether. The reported glycerol conversion was 100%. This GTBE mix was then added to commercial EN 590 European diesel at a concentration of 10 vol%. Additionally, a 40-vol. % biodiesel-diesel blend (B40), having roughly the same oxygen content, was prepared. Both of these blends were used in the engine experiments, together with the commercial diesel fuel as a reference. The results showed that, compared to diesel, the GTBE-diesel blend achieved a reduction in soot emissions, but it resulted in an increase in both THC and CO emissions. However, these increases were not significant. The NO_x_ emissions were slightly lower. Moreover, the cylinder pressure and the heat release rate (HRR) showed no significant deviation from the neat diesel case. Finally, their study included a life cycle analysis (LCA) in which it was shown that the GTBE-diesel blend generated a lower environmental impact than the one produced by neat diesel.

A very similar 2013 study by Frusteri et al. [78], also investigated the influence of the same 10-vol.% GTBE-diesel mix as in the previous study (produced by the same method) on the performance and emissions of the same light-duty single-cylinder diesel engine as in the previous study. As in that previous study, the results showed that the addition of GTBE to diesel did not affect the combustion behavior significantly, as evidenced by the in-cylinder pressure and rate of heat release data. Predictably, GTBE caused a more favorable soot-NO_x_ trade-off, i.e. it produced lower soot emissions for a given NO_x_ level. Moreover, the THC emissions were decreased, whereas the CO emissions increased slightly.

In 2015, Beatrice et al. [79] published an article describing a detailed analysis of the impact of blends of glycerol ethers and diesel fuel on the combustion and emissions on a metal engine, as well as on an optical one. The fuels used were made up of blends of GTBE in fossil diesel at the relatively hight concentrations of 10 and 20 vol%. Both metal and optical engines were light-duty single-cylinder engines, with displacements of 477 and 522 cm^3^, respectively. The test matrix was comprised of selected steady-state operating points of the New European Driving Cycle (NEDC). The metal engine results showed a similar performance between the diesel-GTBE blends and fossil diesel. Moreover, the presence of GTBE resulted in decreased PM emissions, as evidenced by the measured particle size distributions. Also, GTBE caused a shift in the size of the exhaust particles towards smaller diameters. The emissions of NO_x_, THC, and CO did not change significantly, although an increase in aldehyde emissions (formaldehyde and acetaldehyde) was observed, especially at light engine loads. Finally, the optical engine experiments showed that GTBE caused lower soot formation and lower soot peaks for the 20 vol% blend, due to its oxygen content.

### 4.2 Other types of glycerol ethers

In a 2009 patent, Kousemaker and Thiele [80] described a method for producing ethers of glycerol acetals or ketals, to be used as fuel additives for diesel fuels, gasoline, biodiesel, in concentrations up to 30 vol%. As an example, the patent describes the production of solketal, by reacting glycerol with acetone, followed by etherification with isobutylene to form the compound called solketal *tert*-butyl ether (STBE). The rationale behind the additional etherification is to remove the hydroxyl group still present, by substituting it with an alkoxy group, thus increasing the compound’s hydrophobicity, oxidation stability, and heating value [41]. STBE can also be obtained in the other direction, through the ketalization of mono-GTBE with acetone, as described by Samoilov et al. [41]. Monbaliu et al. [81] developed a continuous-flow process for the production of STBE, based on glycerol ketalization with acetone followed by solketal etherification with TBA using sulfuric acid as catalyst. STBE has the potential for becoming a promising fuel additive. According to the work of Tanugula, the addition of STBE in diesel fuel, in concentrations not larger than 5 vol%, caused a reduction in the emissions of NO_x_, CO, and soot [82].

In the first part of the above-mentioned study by Samoilov et al. [83], solketal was compared to its methyl ether, solketal methyl ether (SME), regarding their physicochemical characteristics and their effect on gasoline blending properties, such as volatility oxidation stability, gum formation, and octane numbers. SME was prepared by the methylation of glycerol with methanol followed by the ketalization with acetone. Both solketal and SME were blended with gasoline in concentrations of 1, 2.5, 5, 7.5, and 10 vol%. When compared to solketal, the authors pointed out that SME exhibits lower density, lower boiling point, and higher heating value. In addition, the methylation of solketal’s hydroxyl group causes SME to have lower miscibility with water, an important feature, since high hydrophilicity can cause the extraction of gasoline components with water accumulating in the bottom of fuel tanks and reservoirs. Also, the addition of either solketal or SME to gasoline caused a decrease in oxidation stability, which was worse for SME, and the gum formation decreased with solketal, whereas it significantly increased in the case of SME; moreover, the addition of solketal increased the octane number of the blend (the blending RON reached a peak of 111 at a concentration of 5 vol%), whereas SME caused it to decrease (the mean blending RON was around 52–62). Finally, the authors investigated the effect on the anti-knocking rating of solketal admixtures to conventional gasoline oxygenates: ethanol, methyl *tert*-butyl ether (MTBE), and *tert*-amyl methyl ether (TAME).

The etherification of glycerol with ethanol, was investigated by Pinto et al. [84]. The objective of their study was to produce the glycerol ethyl ethers and use them as biodiesel cold-flow improvers. Their method achieved a glycerol conversion of 96% and 80% selectivity to ethyl ethers. The resulting ether mixture (comprised of 65% mono-ethers, 19% di-ethers, and 16% tri-ether) was blended to both soybean and tallow biodiesel, in concentrations of 0.5–1.0 vol%. According to their results, the addition of the glycerol ethyl ethers decreased the cloud point of the biodiesel by 2 °C and 4 °C, for soybean and tallow biodiesel, respectively, whereas the measured reduction in pour point for soybean and tallow biodiesel was 5 °C and 3 °C, respectively.

The 2017 work by Saengarun et al. [85] describes the production of propyl and butyl ethers of glycerol and their effect on fuel properties. The propylation and the butylation of glycerol was carried out by reacting glycerol with propylene and 1-butene, respectively, over three types of commercial heterogeneous acidic catalysts. The propylation reaction produced a mixture of mono- and di-propyl glycerol ethers, collectively called PGEs. In addition, neat tri-propyl glycerol ether (TPGE) was also produced. Similarly, the butylation reaction yielded a mixture of mono- and di-butyl glycerol ethers, called BGEs. No tri-butyl ether was produced. These ethers were evaluated as both cold flow improvers for biodiesel blends and as octane boosters for gasoline. For the cold flow experiments, the propyl and butyl ethers were added to blends of palm biodiesel and diesel, ranging from B2 (2 vol% biodiesel) to B100 (neat biodiesel). The ethers were added in a wide range of concentrations, from 0.1 to 10 vol %. The results showed that the cloud points of the biodiesel blends decreased with increasing ether concentrations. In the case of PGEs, the reduction in cloud point reached a maximum of 4–7 °C, at ether concentrations of 7–10 vol%. When TPGE was added to the biodiesel blends, the maximum cloud point reduction was 3–7 °C, at concentrations of 7–10 vol%. In the case of BGEs, the reduction in cloud point reached a maximum of 7–13 °C, also at ether concentrations of 7–10 vol%. In the octane number experiments, each glycerol ether, plus ethanol and MTBE were blended with gasoline in concentrations of 10 vol%. According to the results, the research octane number (RON) of neat gasoline (81.1) was increased by 0.5 and 1.0 point when PGEs and TPGE were added, respectively. A maximum increase in RON was observed with the addition of MTBE. Regarding the motor octane number (MON) of gasoline (91.0), the addition of TPGE and PGEs increased it by 2.6 and 3.0 points, respectively. The maximum increase in MON, 7.1 points, was observed when the BGEs were added to gasoline.

The production of glycerol dimethoxy ethers (GDMEs) and glycerol trimethoxy ether (GTME) to be used as biodiesel and diesel fuel additives was investigated by Chang et al. [86]. In their study, the methylation of glycerol was achieved under normal atmospheric pressure conditions using dimethyl sulfate (DMS), producing a mixture of 20 wt% GDMEs and 80 wt% GTME. A conversion of glycerol of 93.5% and an initial combined yield of GDMEs and GTME of 71.2% were obtained using sodium hydroxide as catalyst, at a reaction temperature of 343 K and a reaction time of 24 h. However, at the end of the reaction, the combined yield of GDMEs and GTME fell to 34.5%.

In addition to the ethers described above, the literature also contains studies dedicated to the preparation of other types of glycerol ethers with potential to be used as fuel additives. These include the ethers obtained by reacting glycerol with ethanol [87], n-butanol [88,89], benzyl alcohol [90,91], 1-pentanol, 1-hexanol, 1-octanol, and 1-dodecanol [88,92].

## 5.0 Glycerol esters (glycerol acetates)

**Fig. 5:**
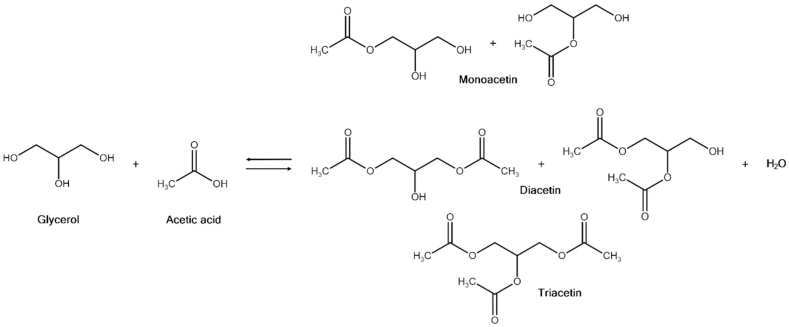
The acetylation of glycerol with acetic acid.

Another way of producing glycerol-derived additives is to react it with carboxylic acids to form esters. As an example commonly found in nature, triglycerides (vegetable oils and animal fats) can be thought of as being the product of the reaction of glycerol with long-chain fatty acids. For fuel additive applications, a typical conversion pathway is the acid-catalyzed esterification of glycerol with acetic acid or acetic anhydride to yield glycerol acetates (also called acetins); this reaction is illustrated in Fig. 5. Depending on the extent of the reaction, three components are formed: monoacetin, diacetin, and triacetin [also known as monoacetylglycerol (MAG), diacetylglycerol (DAG), and triacetylglycerol (TAG), respectively]. Among these, triacetin (TAG) is particularly suited as a fuel additive—usually as cold flow improver or octane booster—due to its good solubility in hydrocarbons, which is caused by the elimination of all three of glycerol’s hydroxyl groups. A 2016 patent by Puche [93], extensively describes a process for producing triacetin and alkyl esters of fatty acids, to be used as octane boosters for gasoline or cold-flow improver for diesel fuels.

The aforementioned work by García et al. [44] also investigated the impact of triacetin blending into B20 diesel (fossil diesel containing 20 vol% biodiesel) and into B100 (neat biodiesel), in concentrations of 5, 10, and 15 vol% concentrations. Their results show that, when triacetin was added to B20, in general, the oxidation stability stayed roughly the same, the density of the blend increased, the viscosity remained roughly the same, the flash point increased, and the cold filter pour point (CFPP) tended to increase. Different results were reported when triacetin was added to neat biodiesel (B100). In this case, regardless of concentration, triacetin increased the oxidation stability of the blend, increased the blend’s density, slightly increased viscosity and decreased the flash point. Perhaps most importantly, triacetin caused a decrease of up to 6 °C in CFPP, confirming its efficacy in improving the cold flow properties of biodiesel.

A new process for catalyst-free biodiesel production was presented by Saka and Isayama in their 2009 article [94]. In their investigation, the triglycerides reacted with supercritical methyl acetate, without any catalyst, at a temperature of 350 °C and a pressure of 20 MPa. This reaction produces fatty acid methyl esters (FAME) and triacetin at a molar ratio of 3:1, respectively (4:1 on a weight basis, corresponding to 20 wt% triacetin). In addition, they added triacetin to methyl oleate (i.e. oleic acid methyl ester) at a 20 wt% concentration in order to investigate the former’s impact on the blend’s properties. The results showed that the addition of triacetin did not have a significant influence on the blend’s viscosity, cold filter plugging point, and flash point. However, it decreased the cetane number of methyl oleate (from 86.3 to 64.5).

Casas et al. [95], in 2010, published an article on the influence of the blending of triacetin on biodiesel’s properties, such as density, viscosity, cloud point, cold filter plugging point, cetane number, heating value, distillation curve, and flash point. In their study, up to 20 wt% triacetin was blended with biodiesels produced from vegetable oils of different feedstocks, namely palm, soybean, sunflower, high-oleic sunflower, and rapeseed. According to their results, the addition of triacetin caused an increase in the blends' density and viscosity, whereas their flash point, heating value, cetane number, cloud point, and pour point decreased when triacetin was added. The cold filter plugging point (CFPP) was increased only when triacetin was added to palm biodiesel; in the case of rapeseed biodiesel, the CFPP decreased instead. Finally, the addition of triacetin did not affect the distillation temperature, expressed as atmospheric equivalent temperature (AET, 90% recovered).

A 2015 study by Lacerda et al. [96] investigated the effect of blending triacetin into fossil diesel fuel and biodiesel on fuel properties and on the exhaust emissions from a small genset. In their experiments, triacetin was blended into fossil diesel fuel and neat biodiesel (B100) in concentrations of 5 and 10 vol%. The results showed that the addition of triacetin caused a decrease in viscosity for both fossil diesel and biodiesel. In addition, triacetin increased the density of both diesel and biodiesel, though the values remained within the range specified by the fuels' standards, EN 590 and EN 14214, respectively. Regarding exhaust emissions, when triacetin was added to either fossil diesel or biodiesel, regardless of concentration, the NO_x_ values remained roughly unchanged, the CO emissions were decreased and the smoke opacity was also decreased.

A 2020 article by Herrada-Vidales [97] describes a procedure to purify crude glycerol from biodiesel production for the subsequent reaction with acetic acid or acetic anhydride to yield triacetin, to be used as octane booster. Triacetin was then compared to both ethanol and MTBE as the standard octane boosters. Blends of triacetin with gasoline in concentrations of 5 and 10 vol% were prepared. In addition, ethanol and MTBE were mixed with gasoline at 5 vol% and 10 vol%, respectively. According to the results, the gasoline blend with 10 vol% triacetin gave comparable performance as the 10 vol% MTBE blend, whereas the octane-boosting ability of the 5 vol% triacetin blend was slightly inferior, when compared to the 5 vol% ethanol blend. These results demonstrated the potential of triacetin as an octane booster when compared to MTBE.

Finally, I n a recent study by Tomar et al. [98], fuel samples containing different amounts of gasoline, methanol, and triacetin were prepared and compared to neat fossil gasoline. The response surface methodology (RSM) was used to optimize engine operating parameters and the results showed that the fuel blend containing 90.7 vol% gasoline, 5 vol% methanol, and 4.3 vol% triacetin gave the best results, based on brake thermal efficiency and exhaust emissions.

## 6.0 Conclusions

The development of fuel additives is one important example of turning the waste glycerol from the biodiesel industry into value-added products, contributing to the industry’s sustainability. There are several different chemical pathways through which waste glycerol can be utilized to produce a number of such fuel additives; according to the chemical group to which they belong, such compounds can be broadly classified into three major categories: acetals/ketals, ethers, and esters (acetates). One common feature of the different additive types is that they can be produced from widely available and inexpensive chemicals, such as formaldehyde, acetone, and acetic acid. In this way, the present work aims to present a concise review of the most common and relevant types of glycerol derivatives that can be used as additives.

Regardless of the chemical category to which it belongs, a glycerol-derived additive typically serves three different functions, depending on the fuel it is blended into: it can act as a cold-flow improver, for biodiesel; an octane booster, for gasoline; and an oxygenate for PM reduction, in diesel fuels. Moreover, the amount of additive to be blended into a given fuel is usually limited to low concentrations, to result in a drop-in fuel, so that the final blend will still conform to the applicable fuel standard (such as EN 590 for diesel fuel, EN 14214, for biodiesel, and EN 228, for gasoline).

Glycerol acetals and ketals are commonly found in the literature. The most prominent member of this category is the compound called solketal, the product of the reaction between glycerol and acetone. It has mostly been investigated as a biodiesel cold-flow improver and as a gasoline octane booster.

The glycerol *tert*-butyl ethers (GTBE), arguably the most commonly investigated types of glycerol-derived fuel additives, are typically obtained by reacting glycerol with either *tert*-butanol or isobutylene. There is a fairly large amount of literature on GTBE and they have been used as biodiesel cold-flow improvers, gasoline octane boosters, and diesel oxygenates. Depending on its conditions, this reaction produces three compounds, namely mono-GTBE, di-GTBE, and tri-GTBE. The second compound represents a compromise between solubility in hydrocarbons and cost, so it is often the target component of GTBE production.

The glycerol acetates (acetins) are the product of the reaction between glycerol and acetic acid or acetic anhydride. As in the case of GTBE, glycerol acetins are comprised of three compounds: monoacetin (also called triacetylglycerol, MAG), diacetin (diacetylglycerol, DAG), and triacetin (triacetylglycerol, TAG). Due to its solubility in hydrocarbons, and due to the fact that it can be produced with 100% selectivity, triacetin is commonly the compound of choice for fuel applications. Like solketal, it has mostly been investigated as a biodiesel cold-flow improver and as a gasoline octane booster.

Other less-known compounds, like solketal *tert*-butyl ether (SBTE), solketal methyl ether (SME), fatty acid glycerol formal ester (FAGE) [99,100], and glycerol carbonate (GC) [101] (the last two not covered in the present work), have also been proposed as fuel additives, but the amount of literature dedicated to these compounds is still small, when compared to the main types described above.

According to the results reported by the studies described in this work, glycerol derivatives were effective in improving the relevant properties of the base fuels, such as gasoline octane rating and biodiesel pour point and cold-filter plugging point. In addition, soot (smoke) emissions were reportedly decreased when these compounds were added to diesel fuels. However, it should be noted that the addition of glycerol derivatives into a given fuel may cause some of the fuel’s properties, such as flash point or density, to be outside of the limits specified in the relevant standard (e.g. EN 228 for gasoline, EN 590 for diesel, or EN 14214 for biodiesel). That’s one major reason the amounts to be blended into the base fuel should be small enough, so that the final blend can be treated as a “drop in” fuel.

Even though there is a considerable body of literature on the main types of glycerol-derived fuel additives, there is still a relative lack of studies on some of their applications, such as glycerol acetals/ketals and acetates being used as diesel fuel oxygenates, rather than as biodiesel cold-flow improvers or gasoline octane boosters. In addition, even though all of these glycerol derivatives are typically used in low concentrations (e.g. 5 vol% or lower), from a scientific point of view it would be also interesting to evaluate them in fuel blends of higher-concentration, such as 10 vol% or even 20 vol%, to further investigate their influence on fuel properties, and on engine performance and emissions.

## Author contribution statement

All authors listed have significantly contributed to the development and the writing of this article.

## Funding statement

This work was supported by the European Union’s Horizon 2020 Framework Program for Research and Innovation, Grant Agreement No. 818310.

## Data availability statement

No data was used for the research described in the article.

## Declaration of interest's statement

The authors declare that they have no known competing financial interests or personal relationships that could have appeared to influence the work reported in this paper.
