# Supplementary material for: A concise review of glycerol derivatives for use as fuel additives
Source: Heliyon. 2023 Jan 18;9(1):e13041. doi: 10.1016/j.heliyon.2023.e13041 (PMC9879789; doi:10.1016/j.heliyon.2023.e13041)
Supplement: Multimedia component 1 [file mmc1.pdf]

RSER Author Checklist Table

Item	Check	Important notes for Authors/Requirement
Article type	Select the single correct article type here and state in brackets the paper word count.<br>• Full-length article (Word count)<br>• Review article (Word count)<br>• Perspectives (Word count)<br>• If the article submitted is by invitation to a Special Issue, also state the Special Issue short name (e.g. VSI: NAME) here.	Papers will be indexed as Full-length articles, Review articles, Retractions, Corrigendum, Addendum or Editorials as explained above in this GFA.
Manuscript	This is the 'entire' article that the reviewers and authors will assess. It is used later to prepare the published article, so it is important that all details required by the GFA are included.	The manuscript should be a single MS Word file or pdf that includes the cover letter, the RSER Author Checklist table and the paper as per the layout in the GFA.
Cover letter	A maximum of two pages, dated and addressed to the Editors stating the name and affiliation of the authors, it should state the following clearly;<br>• Title paper, key findings and why novel and meets the journal scope,<br>• Article type and if relates to a conference special issue.<br>• Any details relating to elements of the work already published as a Preprint/Archiv/Working paper/conference paper etc. or as a thesis or other with a precise explanation,<br>• Any details of funding agencies etc.,<br>• Provide a declaration of interest,<br>• List any recommended reviewers,<br>• The corresponding author must sign the Cover letter as the person held responsible for all aspects of the paper during and after the publication process.	Note that the role of the corresponding author is very important as they are responsible for the article ultimately in terms of Ethics in Publishing, making sure that the GFA is adhered to, informing readers of any relationships with organisations or people that may influence the work inappropriately as discussed in the GFA, all the content of the article and that the Proof is correct.<br><br>It is very difficult if not impossible to edit a paper once published. Most mistakes in articles occur when corresponding authors are changed after/during acceptance; examples include leaving out acknowledgements of funding agencies and the full and correct author affiliations.
Layout of paper	The elements/headings listed below should appear in the order below in the paper:<br>• Title<br>• Author details<br>• Abstract<br>• Highlights<br>• Keywords<br>• Word Count<br>• List of abbreviations including units and nomenclature<br>• 1.0 Introduction<br>• 2.0 Material and methods<br>• 3.0 Theory/calculation<br>• 4.0 Results<br>• 5.0 Discussion<br>• 6.0 Conclusion<br>• Acknowledgements<br>• List of References	Note read carefully the specific details of each element/heading in this GFA. The main headings i.e. 2.0 to 6.0 can vary from article to article, but all articles must include the title, author details, abstract, keywords, highlights, word count and list of abbreviations on page 1 of the paper.
English, grammar and syntax	State checked yes or no.	The authors must proof read and check their work. This is NOT the role of the editorial team, reviewers or the publishing team. Some guidance on English, grammar and syntax is provided in this GFA below, but it is ultimately the author's responsibility.
Title	State checked and adhered to GFA yes or no.	The title should not include acronyms or abbreviations of any kind. Excessive use of capitals letters should also be avoided.
Author names and affiliations	State checked and adhered to GFA yes or no.	The names of the authors in order of contribution or supervision or seniority depending on the funding agency/field requirements should be presented below the title of the article as follows:<br>Last, First by initial e.g. Name, A.M.[1], Name, P.[2]<br>1 = Institution, Post details, Country<br>2 = Institution, Post details, Country

Corresponding author	Show clearly in the paper, beside the name of the author with an asterix and footnote. Confirm that this has been done.	The corresponding author must be denoted in the article by an asterix superscript beside their name and a footnote, as follows: Joe, B.L..[1, *]<br>* = corresponding author details, blogsjoe@email.ac.uk<br>Note that only one corresponding author can be identified.
Highlights	These should be inserted as requested in the article and uploaded as a separate file.	Details on highlights are in the GFA below.
Graphical abstract	State if attached, yes or no.	Note submitting a graphical abstract is at the discretion of the authors. It this is not required by RSER.
Copyright	State checked and adhered to GFA yes or no<br>List here any tables, figures, graphs or other images that required copyright.	Authors are responsible for arranging copyright for any already published images, figures, graphs and tables borrowed from third parties. Citing a source is not enough, in fact this is an Ethics in Publishing issue. Guidance on arranging copyright is provided in the GFA below.
Referencing style	State checked and adhered to RSER preferred style, yes or no.<br>State that all	The preferred journal style is Vancouver (i.e. [1], [2] etc., see details on using in this GFA. All references must be numbered chronologically starting at 1 in square brackets in the paper and the list of references. All references mentioned in the Reference List are cited in the text, and vice versa.
Single column	State checked and adhered to GFA yes or no.	Note an article submitted in two column format will be automatically rejected.
Logos/emblems etc.	State checked and adhered to GFA yes or no.	A paper must NOT be submitted with Elsevier logos/layout as if already accepted for publication.
Embed graphs, tables and figures/other images in the main body of the article	State checked and adhered to GFA yes or no.	Although you will be required to submit all images, images MUST appear embedded in the main body of the article where they are to appear in the final published article.
Figures/Graphs/other images	State checked and adhered to GFA yes or no.	Any captions for graphs should be below the graph in the Manuscript. Note that all figures must be individually uploaded as separate files in the correct format, check format requirement in this GFA. Ensure all figure citations in the text match the files provided.
Tables	State checked and adhered to GFA yes or no.	Any captions for tables should be above the graph in the Manuscript. Note that all figures must be individually uploaded as separate files in the correct format, check format requirement in this GFA. Ensure all table citations in the text match the files provided.
Line numbering	State checked and adhered to GFA yes or no.	RSER journal uses automatic line numbering, so authors must submit their source files without line numbers.
Acknowledgements	State read guidance on Acknowledgements in GFA and included, yes or no.	The questions authors need to ask themselves, when preparing their acknowledgement are as follows:<br>• Was this work funded by a government agency, industry or other philanthropic organisation? If yes, the corresponding author must check and include any grant/award/funding details.<br>• Were any data sources, models, images used or provided by others, who did not contribute to the article? If yes, then it is to good practice to name and thank them individually.<br>• Did any colleagues, friends or family proof read your work? If yes then it is also polite to mention them.
Ethics in Publishing	State checked carefully by all the authors named on the paper.	It is vital that all authors read our requirements for Ethics in Publishing. Once your name is on the article you all are responsible for any plagiarism issues. Note that a corresponding author must email the Editor in Chief to get approval for any changes in authorship before any Proof is finalised. A change in name of the corresponding author must also be done with the written consent of the author and the Editor in Chief nominated by the existing corresponding author.
Ethical Statement	Upload an Ethical Statement or alternatively state in the Cover Letter.	Read details in this GFA,
